# Patient Confidential Data Hiding and Transmission System Using Amplitude Quantization in the Frequency Domain of ECG Signals

**DOI:** 10.3390/s23229199

**Published:** 2023-11-15

**Authors:** Shuo-Tsung Chen, Ren-Jie Ye, Tsung-Hsien Wu, Chun-Wen Cheng, Po-You Zhan, Kuan-Ming Chen, Wan-Yu Zhong

**Affiliations:** 1 Department of Medical Informatics, Chung Shan Medical University, Taichung 40201, Taiwan; shough34@yahoo.com.tw (S.-T.C.); s1058029@gm.csmu.edu.tw (C.-W.C.); s1058038@gm.csmu.edu.tw (P.-Y.Z.); s1058015@gm.csmu.edu.tw (K.-M.C.); s1058006@gm.csmu.edu.tw (W.-Y.Z.); 2 Department of Information Center, Chung Shan Medical University Hospital, Taichung 40201, Taiwan; 3 Graduate School of Applied Chinese Studies, National Yunlin University of Science and Technology, Yunlin 64002, Taiwan; 4 Bachelor’s Program in Business Management, Fu Jen Catholic University, New Taipei City 242062, Taiwan

**Keywords:** transform domain, ECG, amplitude, non-linear, hiding state, switch, PSO

## Abstract

The transform domain provides a useful tool in the field of confidential data hiding and protection. In order to protect and transmit patients’ information and competence, this study develops an amplitude quantization system in a transform domain by hiding patients’ information in an electrocardiogram (ECG). In this system, we first consider a non-linear model with a hiding state switch to enhance the quality of the hidden ECG signals. Next, we utilize particle swarm optimization (PSO) to solve the non-linear model so as to have a good signal-to-noise ratio (SNR), root mean square error (RMSE), and relative root mean square error (rRMSE). Accordingly, the distortion of the shape in each ECG signal is tiny, while the hidden information can fulfill the needs of physiological diagnostics. The extraction of hidden information is reversely similar to a hiding procedure without primary ECG signals. Preliminary outcomes confirm the effectiveness of our proposed method, especially an Amplitude Similarity of almost 1, an Interval RMSE of almost 0, and SNRs all above 30.

## 1. Introduction

An electrocardiogram (ECG) shows the human heart’s electrical activity as a basis to investigate heart disease and diagnose a cardiovascular anomaly. Accordingly, an ECG is a paramount biosignal to be secured and transmitted in a hospital network. For this goal, it is necessary to apply information-hiding techniques on the ECG to preserve the patient’s information. Related research is still an important issue. Engin [1] proposed a simple data-hiding method for ECG signals. However, their method was not blind. Zheng and Qian [2,3] proposed a wavelet-based ECG data-hiding method of a non-QRS complex to ensure the replacement of undistorted ECG signals. Kuar and colleagues [4] proposed a blind hiding method to warrant the secure spreading of ECG signals in wireless networks. Ibaida [5] used an improved least significant bit (LSB) watermarking method to embed the patient’s biomedical information into the ECG signal while ensuring the integrity of the patient’s ECG. Nevertheless, the choice of an immersing position is sophisticated [6].

In [7,8], the authors applied a quantization watermarking technique to ECG signals in a wavelet domain. Nevertheless, this method was blind. However, the quality of each ECG signal embedded with a watermark decreased when the embedding strength increased. Moreover, Zhou [8] proposed a blind recognition model of a single-channel electromyography. Dey et al. [9] embedded a reversible binary watermark into photoplethysmo-graphic (PPG) signals and then extract the watermark by an error-prediction algorithm. The same laboratory [10] inserted the binary watermark image into the electrocardiogram signal and then proposed a new session-based blind watermarking scheme. However, methods [9] and [10] were not blind. In [11,12], a single-coefficient quantization in the transform domain applied digital watermark encryption technology to the ECG for the protection of the patient’s information and competence. Based on this method, the changes in PQRST complexes and magnitude in the ECG signal are trivial. Jero et al. [13,14] applied curvelet transforms to identify coefficients that keep important data regarding diagnosis. The originality of their paper is to propose a curvelet transform of the ECG steganography, adaptive selection of a watermarking location, and a new algorithm of the selecting threshold. In [15], the authors proposed a novel time-frequency watermarking scheme with an adaptive lead-independent beat-to-beat data repository plan. In [16], authors embedded the information obtained from the patient’s data into the ECG signals based on curvelet transform. It is a new method to make the hidden message robust against image-processing attacks. In [17], the authors integrated the watermarking and compression in Fourier domain for electrocardiogram (ECG). In [18], authors proposed an ECG watermarking scheme based on redundant discrete wavelet transform (RDWT) and singular value decomposition (SVD). First, the ECG signal is adjusted into a 2-D matrix by the Pan–Tompkins algorithm. Then, the hybrid of RDWT and SVD is to conceal the patient data and logo image in the 2-D ECG image. Sanivarapu et al. [19] embedded the patient’s data with a QR image into the ECG in the wavelet domain. Initially, they adjusted the ECG signal into a 2-D ECG image by applying the Pan–Tompkins algorithm and applying wavelet transform to deteriorate the 2-D ECG image. Then, they applied QR decomposition to the QR image to reduce the detail coefficient of the wavelet to obtain the hidden information. In [20], the authors proposed an ECG signal watermarking scheme based on multiple embedding strength (MES), which is optimized by a hunger games search (HGS) algorithm. The scheme maintained the imperceptibility–robustness trade-off. Table 1 lists the domain, solution, fining, and gap for these references.

In this study, we develop a new bio-information hiding method on the ECG signals by using the MIT-BIH arrhythmia database [21,22] to protect patients’ information and competence. Firstly, we develop an amplitude optimization model with a hiding state switch in the transform domain to hide patient information in ECG signals. In the model, we consider the optimization of the SNR with respect to two hiding equations to maximize the feature of the hidden ECG signals. Next, we apply particle swarm optimization (PSO) to solve the optimization problem in the proposed non-linear model in order to obtain best signal-to-noise ratio (SNR), root mean square error (RMSE), and relative root mean square error (rRMSE). Accordingly, the shape distortion in each ECG signal is extremely small. The removal of hidden information is reversely similar to the hiding procedure without the original ECG signals. In experiments, we evaluate the relationship between the hiding strength Q and SNR, the hiding strength Q and RMSE, and the hiding strength Q and similarity. The results of our experiment confirm the efficacy of this proposal.

This paper is composed of five main parts. Part 1 gives an introduction to the background knowledge. Part 2 reviews some preliminaries. Part 3 mainly introduces the proposed hiding method including an optimization model and its solver PSO. Part 4 demonstrates our outcome results and discussion in this study. Part 5 lists our conclusions.

## 2. Preliminaries

In this part, we recall the waveform of the ECG and point out its fundamental requirement when performing information hiding.

### 2.1. ECG Signal

Electrocardiography is a technique that uses repeated cardiac cycles to record the electrophysiological activity of the heart in units of time. It works by plotting voltage versus time of the heart’s electrical activity using electrodes placed on the skin. These electrodes detect small electrical changes caused by depolarization and repolarization of the heart muscle during each cardiac cycle (heartbeat). A cardiac cycle is divided into P, Q, R, S, and T complexes. The electrocardiogram contains three major components: the P wave represents atrial depolarization; the QRS complex represents ventricular depolarization; and the T wave represents ventricular repolarization.

### 2.2. Discrete Wavelet Transform (DWT)

**Figure 1:**
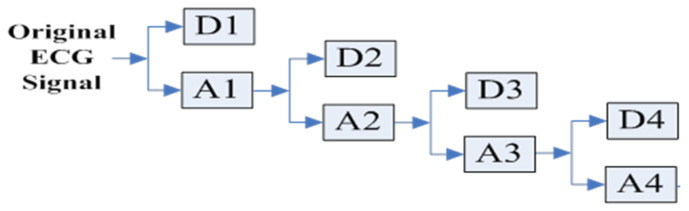
The 4-level DWT decomposition.

The DWT is a scaling method which translates the mother wavelet ψ(x). The normalized wavelets obtained by scales are defined as φi,n(t)=2i2hiφ(2it−n), ψi,n(t)=2i2giψ(2it−n), where i and n stand for scale and translation parameters, and $h_{i}$ and $g_{i}$ stand for low-pass and high-pass filters. In order to decompose the input signal into multiple non-overlapping multi-resolution sub-bands, including high-frequency sub-bands and low-frequency sub-bands, we choose orthogonal wavelet basis functions to expand the coefficients. For example, Figure 1 shows a 4-level Haar DWT with orthogonal wavelet bases.

### 2.3. Discrete Fourier Transform (DFT)

Discrete Fourier Transform (DFT) is a discrete form of Fourier transform in both the time domain and frequency domain, converting the time domain samples of the signal into the frequency domain samples of its DTFT. Since it contains a certain amount of quantity information, this may be accomplished by computers with numerical algorithms or customized tools. The achievements apply structured fast Fourier transform (FFT) algorithms in a way that “FFT” and “DFT” are usually utilized mutually. The DFT has been an important discrete transform used in many practical applications to perform Fourier analysis. In digital signal processing, a function is any time-varying quantity or signal that is sampled over a finite time interval, such as a radio signal or a daily temperature value. In image processing, samples can be pixel values along rows or columns of a raster image. DFT can also be used to solve equations such as partial differential equations.

### 2.4. Discrete Cosine Transform (DCT)

The discrete cosine transform (DCT) is a finite sequence of data samples represented by the sum of cosine functions oscillating at different frequencies and is widely used in signal processing and data compression. In addition, DCT is also used in most digital media, including digital imaging, digital video, digital audio, digital television, and digital broadcasting. In particular, DCT is a Fourier-related transform interchangeable to DFT applying barely real numbers.

### 2.5. Particle Swarm Optimization (PSO)

Particle swarm optimization (PSO) was proposed by Eberhart and Kennedy in 1995. It was inspired by observing the social behavior of foraging birds and applied it to search for solutions to optimization-related problems. The search method is to use a group of potential solutions called particles to find the best solution position in the multi-dimensional solution space. Each time a particle moves, it will refer to the best solution position it has found in the past and the past best solutions of all particles. The position then determines the movement direction and distance. Therefore, PSO is a method with group intelligence and a new branch of evolutionary computing. However, PSO is not guaranteed to find an optimal solution exactly [23,24,25,26,27].

## 3. Proposed System

This part shows the recommended amplitude-quantization technique for hiding patient information in ECGs in the transform domain.

### 3.1. Information Hiding and Detection

**Figure 2:**
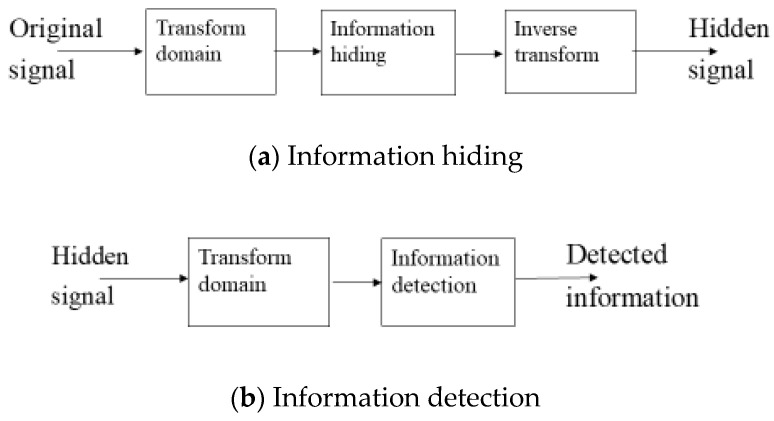
Block diagram for information (**a**) hiding and (**b**) detection.

Information hiding means embedding certain data into a digital carrier comprising signal, imaging, video, document, software, etc. This will not reduce the performance of the initial carrier and is difficult to detect or catch by human visual and auditory perception systems. The block diagram for information hiding in the transform domain is given in Figure 2.

The ECG diagnosis is based on the waves of the PQRST. Accordingly, it is necessary to keep these waveform shapes as we include data in the ECG signals or execute their compression. Presumably, the signal of the ECG is changed when the patient’s confidential information is embedded. The modification is normally defined as distortion. With the intention of decreasing the ECG signal distortion, we scrutinize to maximize the SNR during information hiding.

The information hiding and detection proposed in this study is introduced as follows. In information hiding, we first denote $S=\left\{s_{1},s_{2},\cdots ,s_{n}\right\}$ to be one segment of a patient’s ECG signal. Subsequently, we transform the DWT, DCT, and DFT separately on the ECG signal $S=\left\{s_{1},s_{2},\cdots ,s_{n}\right\}$ to obtain transform-domain coefficients $C=\left\{c_{1},c_{2},\cdots ,c_{n}\right\}$ in DWT, DCT, and DFT so that the binary bits $B=\left\{0,1\right\}$ can be hidden in the amplitude performed by the coefficients $C=\left\{c_{1},c_{2},\cdots ,c_{n}\right\}$ as follows.

(1a) $$\mathrm{Maximize}\;10\log\left[\frac{\sum_{i=1}^{n}{\left|c_{i}\right|}^{2}}{\sum_{i=1}^{n}{\left(\left|{\tilde{c}}_{i}\right|-\left|c_{i}\right|\right)}^{2}}\right]$$

(1b) Subject to ∑i=1nc˜i=α∑i=1nciQQ+34Q+(1-α)∑i=1nciQQ+14Q

where $\tilde{C}=\left\{{\tilde{c}}_{1},{\tilde{c}}_{2},\cdots ,{\tilde{c}}_{n}\right\}$ denotes the hidden ECG signal; *Q* is the hiding strength; α=1 or α=0 represents the hiding state of the binary bit “$\beta_{i}=1$” or “$\beta_{i}=0$”.

In information detection, we utilize the formula in (2) for detecting binary bits $B^{*}$ from the DWT, DCT, and DFT coefficient-amplitude $\left\{{\tilde{c}}_{i}^{}\right\}$

(2) B*=1, if ∑i=1Nc˜i−∑i=1Nc˜iQQ≥Q20, if ∑i=1Nc˜i−∑i=1Nc˜iQQ<Q2

### 3.2. Enhance Performance by PSO

In this sub-part, we adopt the solver PSO [20,21,22,23] to find the optimization solutions of ${\left\{{\tilde{c}}_{i}\right\}}_{i=0}^{N}$ the proposed optimization model approximately in (1). We suppose $x_{j,h}(t)$ and $v_{j,h}(t)$ are the position and velocity of the *h*th dimension for the *j*th particle at time *t*. There are two calculating equations:

(3) $$v_{j,h}(t)=v_{j,h}(t-1)+k_{1}r_{1}(x_{j,h}^{*}-x_{j,h}(t-1))+k_{2}r_{2}(x_{h}^{\#}-x_{j,h}(t-1))$$

(4) $$x_{j,h}(t)=x_{j,h}(t-1)+v_{j,h}(t)$$

where $x_{j}^{*}$ and $x^{\#}$ stand for the finest position solution of the *j*th particle with all particles as of time *t*−1; both $r_{1}$ and $r_{2}$ denote random numbers; $k_{1}$ and $k_{2}$ represent the individuality coefficient and sociality coefficient; it is normally set to 2.

From the previous two calculating equations and fitness $10\log\left[\sum_{i=1}^{n}{\left|c_{i}\right|}^{2}/\sum_{i=1}^{n}{\left(\left|{\tilde{c}}_{i}\right|-\left|c_{i}\right|\right)}^{2}\right]$, we itemize the solving solutions of PSO phases by the proposed optimization model as follows.

Phase I. Set the sample size to 20, then give the beginning value arbitrarily for the position and velocity of every particle.

Phase II. Calculate the fitness $10\log\left[\sum_{i=1}^{n}{\left|c_{i}\right|}^{2}/\sum_{i=1}^{n}{\left(\left|{\tilde{c}}_{i}\right|-\left|c_{i}\right|\right)}^{2}\right]$ of the potential position solution for every particle *j*. When the fitness of the position solution is better than the individual position solution in old memory, the individual position solution is updated.

Phase III. Search the novel finest position solution in the whole particle swarm. If the fitness of this position solution is superior to the previous one, then it is upgraded.

Phase IV. If the terminating condition is satisfied, then the PSO is stopped. If not, go to phase V.

Phase V. Use the PSO flowchart to upgrade the velocity and position solution of every particle. Return to phase II in order to proceed.

### 3.3. Flowchart

**Figure 3:**
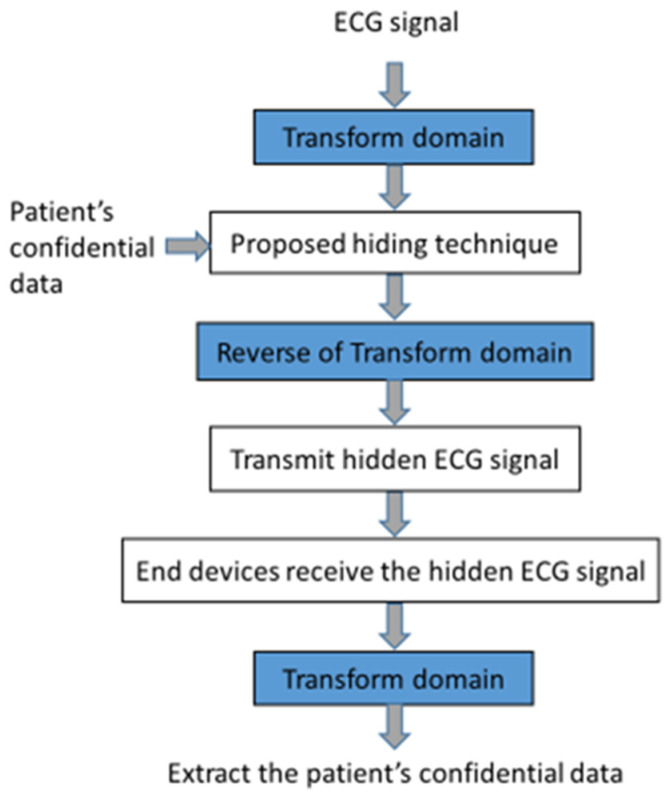
A flowchart of the proposed method.

Figure 3 shows a flowchart hiding the patient’s information in the ECG signal in the transform domain. The transform domain is first applied to ECG signals and then the authentication and patient’s confidential data are hidden in the coefficient-amplitude of the transform domain by the proposed information-hiding method. Finally, we perform an inverse transform to obtain the hidden ECG signals. At the other end, the authentication and patient’s confidential data are conducted after the hidden ECG signals are received and the performing of the transform. Practically, our daily life is full of many other applications.

## 4. Experiments and Discussion

In experiments, we apply the ECG data ID100 to ID105 obtained through the MIT-BIH arrhythmia database [21,22] to examine our suggested solution. The ECG data possess a 360 Hz sampling rate together with a 12-bit binary characterization. Every ECG signal is converted to zero in order to erase the DCT offset. Nevertheless, the settlement of the 12-bit binary characterization is to predict the precise coefficients for hiding the patient’s confidential data. Thus, every signal of ECG with 12-bit binary characteristics is graded to 1 with 16-bit characteristics comparatively. The experimental procedure and results are listed in the following.

First of all, we apply 5-level Haar DWT with orthogonal wavelet bases to degrade an ECG signal to six non-overlapping sub-bands. In order to avoid distorting too much, we hide the binary bits in the multiple low-frequency coefficient-amplitude in the fifth level. The hiding in the DCT and DFT also adopts the low-frequency coefficient-amplitudes, respectively.

Next, we perform the transform domain DWT, DCT, and DFT in every ECG signal of length 4096 selected via 47 datasets from the MIT-BIH arrhythmia database. Study outcomes for the proposed solution in the sample of *N* = 2 are analyzed in the following.

Without loss of generality, presentation of the proposed scheme is examined by the SNR, similarity, and RMSE, as in the following formulas:

(5) $$\mathrm{SNR}=10\log_{10}\left(\frac{\sum_{i=1}^{N}s_{i}^{2}}{\sum_{i=1}^{N}{\left({\tilde{s}}_{i}-s_{i}\right)}^{2}}\right)$$

(6) $$\mathrm{Similarity}\left(S,\tilde{S}\right)=\frac{\sum_{i=1}^{N}s_{i}{\tilde{s}}_{i}}{\sum_{i=1}^{N}{\tilde{s}}_{i}^{2}}$$

(7) $$\mathrm{RMSE}=\sqrt{\frac{1}{N}\sum_{i=1}^{N}{\left({\tilde{s}}_{i}-s_{i}\right)}^{2}}$$

where $s_{i}$ and ${\tilde{s}}_{i}$ indicate theoriginal sample and hidden sample in some ECG signals.

The proposed information detection is similar to the proposed information-hiding procedure. First of all, we transform DWT, DCT, and DFT in the test ECG signals, respectively. Next, we detect the binary bits by the rule in (2).

**Figure 4:**
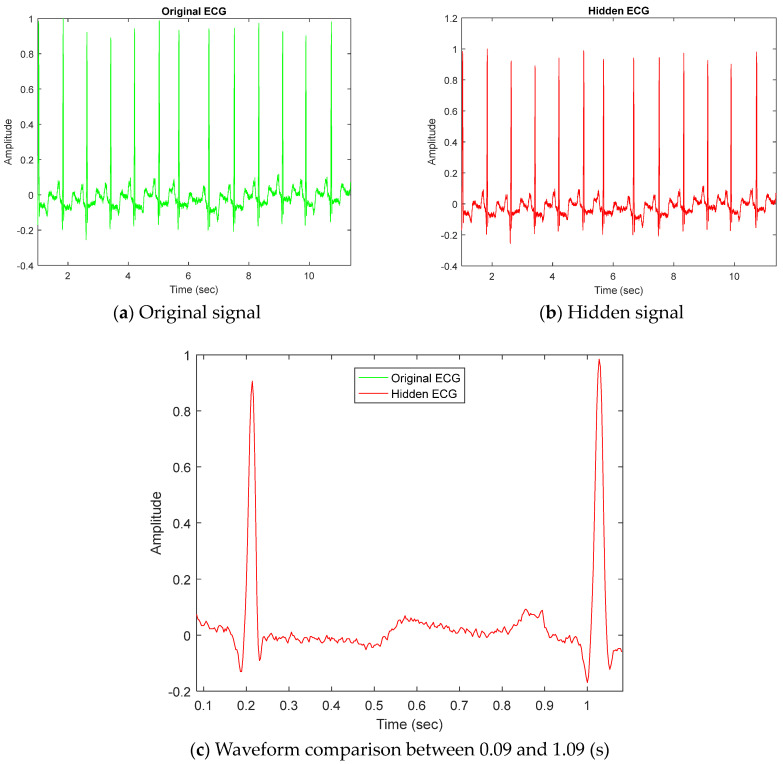
Differentiation of original signals and hidden signals for dataset ID 100.

Each hidden ECG signal has good quality because of the proposed method. For example, Figure 4a,b show the original ECG signal and the hidden ECG signal of dataset ID 100 using DWT lowest-frequency coefficients in a 5-level decomposition. They are similar, as shown in Figure 4c, where the green curve demonstrates primitive ECG signals; in addition, the green curve shows hidden ECG signals by DWT and the quantization size *Q* = 3000.

Moreover, as shown in Table 2, our recommended technique applies PSO (20 particles) to maximize amplitude similarity and SNR but minimize RMSE. SNR is significantly diminished when the quantization size *Q* is escalated. In addition, our technique persists with high quality, small RMSE and high SNR, for each hidden ECG signal under a sufficient hiding capacity when the quantization size *Q* is escalated. Both DFT and DCT also have the same effect on the RMSE and SNR. For extracting the embedded patient information, the average success rate of extraction is about 81.2% since PSO does not guarantee that it can accurately solve the proposed optimization model in Equation (1a,b). Usually, the average success rate of extraction in non-approximation hiding methods is above 90%. For example, the average success rate of extraction in reference [12] is 94.8%. Compared with these methods, the success rate of extraction in our method is lower.

When transmitting data, they is sent to the receiver by the transmission speed of 1 Giga (G) bps. When the quantity of information transmitted is not large enough or during a restriction on transmitting data, information-hiding is recommended.

## 5. Conclusions

By developing an optimization model, we combine coefficient-amplitude quantization with a SNR to propose a new hiding method for a patient’s confidential data in this study. After testing six ECG datasets by using the proposed hiding method, the distinction of the hidden ECG signal and primitive one has an Amplitude Similarity of almost 1, an Interval RMSE of almost 0, and SNRs all above 30, which is tiny and insignificant for physiological detection. In addition, our recommended technique ameliorates the weakness of each hidden ECG signal, which is significantly diminished when the quantization size Q is escalated. However, our proposed method has the limitation of a low extraction success rate. In future work, we will improve the shortcomings of the low extraction success rate.

## Figures and Tables

**Table 1:** List of domain, solution, fining, gap for references [11,12,13,14,15,16,17,18,19,20].

Reference	Domain	Solution.	Fining	Gap.
[11]	transform domain	single-coefficient quantization	In case of embedding strength by SNR = 32, most of amplitude simi-larities are 1, root mean square ap-proaches 0.	For increase in embedding strength, there is decrease in the SNR value.
[12]	transform domain	single-coefficient quantization	In case of embedding strength by SNR = 32, most of amplitude similarities are 1, root mean square approaches 0.	For increase in embed-ding strength, there is decrease in the SNR value.
[13]	transform domain	adaptive selection of watermarking location and threshold selecting for embedding	As the patient data size is increased, the cover signal deteriorates but the Bit Error Rate is zero. The signal deterioration is about 10% when patient data increase 1.5 times.	For increase in watermark size, there is decrease in the PSNR value.
[14]	transform domain	adaptive selection of watermarking location and selecting threshold for embedding	As the patient data size is increased, the cover signal deteriorates but the Bit Error Rate is zero.	For increase in watermark size, there is decrease in the PSNR value.
[15]	transform domain	adaptive lead-independent beat-to-beat data repository plan	(1) The 11th order Symlet is the best among the wavelets tested (2) The watermark and noise similarity of amplitude and numerical distribution are highly affected	Further watermarking destroys the existing watermark.
[16]	transform domain	embed the information of the patient’s data into the ECG signals using curvelet transform	A PSNR value higher than 64 shows the high quality of extracted information. The NC values of all ECG signals are 1 and the SSIM values are close to 1, which indicates high similarity between embedded and extracted information.	Some false positives occur during the watermark embedding process, which not only reduces the quality of the extracted watermark, but also affects the robustness of the image.
[17]	transform domain	ECG watermarking and compression in Fourier domain	The improved SNR proves the denoising ability of the watermark signal.	For increase in embedding strength, there is decrease in the SNR value.
[18]	transform domain	ECG watermarking using the integration of redundant discrete wavelet transform (RDWT) and singular value decomposition (SVD)	The optimal invisibility and robustness result are more effective.	There does not seem to be any guarantee that the encoded binary bits could be recovered using particle swarm optimization (PSO).
[19]	transform domain	embedding factor value is calculated adaptively by harnessing the entropy value of the signal	The embedding factor value is calculated adaptively by harnessing the entropy of the ECG signal. The embedded data can be easily extracted with no distortion.	For increase in embedding strength, there is decrease in the SNR value.
[20]	time domain	multiple embedding strength optimized by hunger games search algorithm	maintaining the imperceptibility-robustness trade-off to obtain PSNR = 57.725 dB	Embedded patient information is less hidden and less robust.

**Table 2:** Experimental results for the three transforms: DWT, DCT, and DFT.

ID	Method	Domain	Q	Amplitude Similarity	SNR	RMSE	Interval RMSE in ECG			
							PR	QRS	ST	QT
	Reference [12]	DWT (Level 5)	400	1	40.85	36.86	0	0	0	0
			1000	1	35.16	67.43	0	0	0	0
			3000	1	24.26	121.52	0	0	0	0
		DFT	400	1	61.13	3.75	0	0	0	0
			1000	1	54.68	8.21	0	0	0	0
			4000	0.99	46.12	28.36	0	0	0	0
		DCT	400	0.99	29.92	197.92	0	0	0	0
			1000	0.98	20.16	435.11	0	0	0	0
			3000	0.81	9.21	2014.6	0	0	0	0
	Proposed	DWT (Level 5)	400	0.99	39.86	51.93	0	0	0	0
			1000	0.99	39.14	49.22	0	0	0	0
			3000	0.99	38.95	49.37	0	0	0	0
		DFT	400	1	34.22	93.15	0	0	0	0
			1000	1	31.81	109.74	0	0	0	0
			3000	1	31.09	105.82	0	0	0	0
		DCT	400	1	47.16	19.88	0	0	0	0
			1000	1	45.94	21.90	0	0	0	0
			3000	1	45.78	23.12	0	0	0	0
101	Reference [12]	DWT (Level 5)	400	1	41.78	36.39	0	0.001	0.002	0
			1000	1	34.67	71.65	0	0	0	0
			3000	1	25.39	141.47	0	0	0	0
		DFT	400	1	60.42	4.12	0	0	0	0
			1000	1	54.37	6.95	0	0	0	0
			3000	1	45.58	29.73	0	0	0	0
		DCT	400	0.99	26.63	203.31	0	0	0	0
			1000	0.98	18.67	441.87	0	0	0	0
			3000	0.78	8.32	1983.4	0	0	0	0
	Proposed	DWT (Level 5)	400	1	33.45	92.56	0	0	0	0
			1000	1	33.16	92.13	0	0	0	0
			3000	0.99	32.89	96.09	0	0	0	0
		DFT	400	0.99	32.25	107.58	0	0	0	0
			1000	0.99	30.82	103.14	0	0	0	0
			3000	0.99	30.19	94.31	0	0	0	0
		DCT	400	1	36.46	70.15	0	0	0	0
			1000	1	35.83	68.63	0	0	0	0
			3000	1	35.47	68.51	0	0	0	0
102	Reference [12]	DWT (Level 5)	400	0.99	44.65	33.94	0	0	0	0
			1000	0.99	37.35	70.16	0	0	0	0
			3000	0.99	27.31	267.17	0	0	0	0
		DFT	400	1	63.18	4.12	0	0	0	0
			1000	1	56.28	7.45	0	0	0	0
			3000	1	47.53	30.28	0	0	0	0
		DCT	400	0.99	28.02	200.19	0	0	0	0
			1000	0.99	21.45	421.37	0	0	0	0
			3000	0.87	13.28	1890.5	0	0	0	0
	Proposed	DWT (Level 5)	400	1	34.28	92.78	0	0	0	0
			1000	1	34.22	95.68	0	0	0	0
			3000	1	33.42	115.24	0	0	0	0
		DFT	400	0.99	28.62	300.25	0	0	0	0
			1000	0.99	26.61	334.68	0	0	0	0
			3000	0.99	26.60	293.24	0	0	0	0
		DCT	400	1	29.57	171.13	0	0	0	0
			1000	1	29.36	170.12	0	0	0	0
			3000	1	29.24	166.77	0	0	0	0
103	Reference [12]	DWT (Level 5)	400	1	40.56	36.07	0	0	0	0
			1000	1	37.65	63.48	0	0	0	0
			3000	1	26.35	270.75	0	0	0	0
		DFT	400	1	63.23	4.07	0	0	0	0
			1000	1	55.76	8.13	0	0	0	0
			3000	0.99	47.12	30.08	0	0	0	0
		DCT	400	0.99	28.23	211.17	0	0	0	0
			1000	0.99	21.62	450.70	0	0	0	0
			3000	0.85	10.21	1980.2	0	0	0	0
	Proposed	DWT (Level 5)	400	1	27.38	258.11	0	0	0	0
			1000	1	26.75	238.72	0	0	0	0
			3000	1	26.48	253.62	0	0	0	0
		DFT	400	0.99	28.32	155.43	0	0	0	0
			1000	0.99	28.54	160.41	0	0	0	0
			3000	0.99	28.61	158.37	0	0	0	0
		DCT	400	1	34.17	96.24	0	0	0	0
			1000	1	34.11	95.84	0	0	0	0
			3000	1	34.25	94.33	0	0	0	0
104	Reference [12]	DWT (Level 5)	400	0.99	41.56	37.21	0.041	0	0	0
			1000	0.99	37.20	68.96	0	0	0	0
			3000	0.99	23.67	260.17	0	0	0	0
		DFT	400	1	62.46	4.37	0.653	0.015	0	0
			1000	1	56.47	8.57	0	0	0	0
			3000	0.99	44.45	30.16	0	0	0	0
		DCT	400	0.99	27.84	202.645	0	0	0	0
			1000	0.99	21.26	431.98	0	0	0	0
			3000	0.86	8.41	1895.5	0	0	0	0
	Proposed	DWT (Level 5)	400	0.99	25.16	317.65	0	0	0	0
			1000	0.99	25.34	327.75	0	0	0	0
			3000	0.99	25.76	322.64	0	0	0	0
		DFT	400	0.99	27.18	166.73	0	0	0	0
			1000	0.99	27.29	167.35	0	0	0	0
			3000	0.99	27.36	166.46	0	0	0	0
		DCT	400	1	31.41	134.54	0	0	0	0
			1000	1	32.26	130.17	0	0	0	0
			3000	1	32.18	128.96	0	0	0	0
105	Reference [12]	DWT (Level 5)	400	1	41.96	34.54	0	0	0	0
			1000	1	39.15	68.28	0	0	0	0
			3000	0.99	24.75	284.63	0	0	0	0
		DFT	400	1	64.46	3.70	0	0	0	0
			1000	1	58.37	7.47	0	0	0	0
			3000	1	46.24	30.21	0	0	0	0
		DCT	400	0.98	29.58	205.58	0	0	0	0
			1000	0.99	22.76	450.82	0	0	0	0
			3000	0.88	9.43	2092.6	0	0	0	0
	Proposed	DWT (Level 5)	400	1	26.83	283.36	0	0	0	0
			1000	1	26.39	297.79	0	0	0	0
			3000	1	26.56	291.29	0	0	0	0
		DFT	400	0.99	25.96	161.36	0	0	0	0
			1000	0.99	25.74	162.85	0	0	0	0
			3000	0.99	25.36	161.46	0	0	0	0
		DCT	400	1	46.58	31.01	0	0	0	0
			1000	1	46.00	31.05	0	0	0	0
			3000	1	46.22	28.54	0	0	0	0

## Data Availability

Data is contained within the article.
